# Artificial Intelligence-Assisted Capsule Endoscopy for Obscure Small-Bowel Bleeding: A Systematic Review of Workflow Gains and the Unmeasured Impact on Patient-Centred Outcomes

**DOI:** 10.7759/cureus.96037

**Published:** 2025-11-03

**Authors:** Abdulkreem Al-Juhani, Amirah A Alzaki, Esraa F Maghrabi, Reem Rambo, Azzah AlGhamdi, Nada A Alanazi, Rodan Desoky, Mahmoud S Desoky

**Affiliations:** 1 Forensic Medicine, Forensic Medicine Center, Ministry of Health, Jeddah, SAU; 2 Surgery, King Abdulaziz University Faculty of Medicine, Jeddah, SAU; 3 Internal Medicine, University College Dublin, Dublin , IRL; 4 School of Medical Science, Universiti Sains Malaysia, Kelantan, MYS; 5 Medicine, King Faisal Specialist Hospital and Research Centre, Jeddah, SAU; 6 College of Medicine, Alfaisal University, Riyadh, SAU; 7 Internal Medicine/Gastroenterology, Sultan Bin Abdulaziz Humanitarian City, Riyadh, SAU

**Keywords:** artificial intelligence, capsule endoscopy, computer‑aided detection, diagnostic yield, grade, obscure gastrointestinal bleeding, reading time, risk of bias, small‑bowel bleeding

## Abstract

Small‑bowel capsule endoscopy (SBCE) is a first‑line diagnostic tool* *after negative bidirectional endoscopy in obscure/suspected small‑bowel bleeding, but reading is time‑intensive. While artificial intelligence (AI)‑assisted review promises faster interpretation and more consistent detection, patient‑level benefits remain uncertain. The aim of this review is to compare AI‑assisted versus conventional SBCE reading in adults with obscure/suspected small‑bowel bleeding, focusing on management‑direct outcomes and, when absent, surrogate outcomes (per‑patient bleeding‑relevant yield; reading time). This was a systematic review following the Preferred Reporting Items for Systematic Reviews and Meta-Analyses (PRISMA) 2020 and Synthesis Without Meta-analysis (SWiM) guidelines. We included randomized/quasi‑experimental trials and prospective comparative cohorts (Tier 1) and retrospective paired re‑readings (Tier 2) published between 2015 and 2025. Primary outcomes included time‑to‑diagnosis/first therapeutic intervention, transfusion, therapeutic yield of balloon-assisted enteroscopy/interventional radiology-guided therapy (BAE/IR), rebleeding/readmission, and costs. Co‑primary surrogates included per‑patient diagnostic yield of bleeding‑relevant lesions (prefer P2) and reading time. Risk of bias was assessed by RoB 2 (Cochrane, London, United Kingdom)/Risk Of Bias In Non-randomized Studies - of Interventions (ROBINS‑I) and certainty by the GRADE (Grading of Recommendations Assessment, Development and Evaluation) approach. The synthesis of the included studies showed eight comparative studies across four platforms (PillCam™ (Medtronic plc, Minneapolis, Minnesota, United Kingdom), MiroCam^® ^(IntroMedic Co., Ltd., Seoul, Republic of Korea), NaviCam® (AnX Robotica Corp, Plano, Texas, United States), and OMOM capsule endoscopy (Jianshan Science and Technology (Group) Co., Ltd, Chongqing, China) met the criteria (Tier 1 = 4; Tier 2 = 4).

No study reported any management‑direct outcome. AI assistance consistently reduced reading time by ~20-58 minutes (relative ~50-92%) and increased throughput ~2-13×, with concordant findings across designs and AI modes. Per‑patient bleeding‑relevant yield was improved with a modern deep‑learning system, approximately equivalent to a newer rapid mode, and lower with an earlier rapid‑review generation, suggesting a technology‑generation effect. Risk‑of‑bias was predominantly moderate; one study had serious risk (positive‑only cohort), and the randomized reading‑allocation trial showed some concerns. Certainty of evidence was moderate for reading‑time reduction, low for per‑patient bleeding‑relevant yield, and absent for management outcomes. In conclusion, AI‑assisted SBCE is operationally superior to conventional reading and diagnostically at least comparable, often better with contemporary deep‑learning, in adults investigated for obscure/suspected small‑bowel bleeding. Whether these efficiency gains improve patient‑centred outcomes remains unproven and requires prospective pathway‑level trials that measure time‑to‑therapy, transfusions, rebleeding, and costs using standardized bleeding‑relevant endpoints.

## Introduction and background

Obscure gastrointestinal bleeding (OGIB) or suspected small-bowel bleeding (SSBB) is a common and expensive indication for small-bowel capsule endoscopy (SBCE) [1,2]; however, its interpretation is labor-intensive and susceptible to reader fatigue due to the scarcity of clinically significant frames within hours of recording. Artificial intelligence (AI)-assisted reading has been developed to prioritize frames, identify clinically significant lesions, and reduce review time, with the potential to expedite diagnostic processes. A multicentre prospective study of adults evaluated for SSBB revealed that deep-learning-assisted reading enhanced per-patient detection of bleeding-relevant lesions and significantly decreased mean reading time, indicating that AI can improve both efficiency and diagnostic yield relative to standard reading [3]. 

Subsequent prospective assessments of rapid-review methodologies that algorithmically mitigate redundancy across several capsule platforms have consistently demonstrated significant time savings while maintaining diagnostic efficacy compared to traditional human review [4-6]. These studies indicate that review durations can be reduced from over an hour to mere minutes without compromising clinically significant detection, implying that AI or algorithmic triage could substantially alleviate reporting backlogs and accelerate subsequent decision-making. Retrospective paired re-readings of bleeding cohorts validate these findings, demonstrating significant reductions in reading time and heightened sensitivity for current bleeding and angiodysplasias when AI-assisted triage is employed as the preliminary assessment prior to a confirmatory human review [7-9]. 

Empirical implementation studies provide significant efficiency improvements with AI-assisted software incorporated into common practice, without a reduction in lesion detection compared to conventional workflows [10].

Notwithstanding these advancements, patient-level management outcomes mostly remain unquantified. In the existing comparative studies, researchers have not evaluated whether AI-assisted reading reduces the time to definitive diagnosis or treatment (such as balloon-assisted enteroscopy or angiography), decreases transfusion needs, enhances therapeutic efficacy, minimizes rebleeding or readmissions, or influences costs. This systematic analysis synthesizes comparative evidence from 2015 to 2025 of AI-assisted versus conventional SBCE reading in people assessed for OGIB/SSBB. The main aim of the review is to ascertain if AI-assisted reading alters clinical treatment and results; secondary aims include measuring the impact on reading duration and inter-reader performance.

## Review

Methodology 

Study Design 

We performed a protocol-driven systematic review following Preferred Reporting Items for Systematic Reviews and Meta-Analyses (PRISMA) 2020 and Synthesis Without Meta-analysis (SWiM) guidelines. The study was not registered in the International Prospective Register of Systematic Reviews (PROSPERO). The a priori review question was: Among adults undergoing SBCE for OGIB/SSBB, does AI-assisted, human-in-the-loop interpretation, compared to traditional human interpretation, enhance clinical management and patient outcomes; if not, does it improve clinically significant diagnostic yield and reading duration?

Eligibility Criteria 

Eligibility was delineated via Population, Intervention, Comparison, Outcomes, and Study (PICOS) design as given below.

Population: adults (≥18 years) examined for OGIB/SSBB (overt or occult) generally following negative or inadequate esophagogastroduodenoscopy (EGD) and colonoscopy; mixed-indication cohorts were permissible if at least 50% had OGIB/SSBB or if OGIB-specific data were obtainable. 

Intervention: any human-in-the-loop AI or algorithmic reading-stage assistance (e.g., deep-learning computer-aided detection (CAde), frame-ranking/rapid modes such as TOP100 or ExpressView, commercial clinical AI suites).

Comparator: traditional comprehensive human analysis of video content without AI pre-screening. 

Outcomes: predetermined primary, management-related outcomes (time to diagnosis or first therapeutic intervention such as balloon-assisted enteroscopy (BAE)/angiography; transfusion exposure; therapeutic efficacy of subsequent operations; rebleeding/readmissions within set intervals; costs/resource utilization). In the absence of these, we employed co-primary surrogates: per-patient diagnostic yield of clinically significant bleeding lesions (prefer Saurin P2) and reading duration (minutes). 

Study designs: a hierarchical framework was employed. Tier 1 comprised randomized or quasi-experimental trials and prospective comparison cohorts (either parallel or paired); Tier 2 encompassed retrospective paired or crossover re-evaluations of identical movies following a predetermined process. 

We rejected papers that utilized only algorithm-based test sets without human comparators, single-arm series, studies focused solely on pediatrics, and non-SB capsules unless the findings of SBCE/OGIB were distinguishable. Articles published between January 1, 2015, to September 18, 2025, were evaluated; applicable to any setup and any SBCE platform. No linguistic restrictions were imposed during the search; non-English papers with English abstracts were evaluated.

Study Selection and Strategy 

Information sources included MEDLINE (Medical Literature Analysis and Retrieval System Online; Ovid), Embase (Excerpta Medica dataBASE), Cochrane Controlled Register of Trials (CENTRAL), Web of Science Core, and Scopus, trial registries (ClinicalTrials.gov, International Clinical Trials Registry Platform (ICTRP), and conference abstracts from Digestive Disease Week (DDW), United European Gastroenterology Week (UEGW), European Society of Gastrointestinal Endoscopy (ESGE) Days, and British Society of Gastroenterology (BSG) (2015-2025). A librarian optimized search tactics by integrating terms related to capsule endoscopy, OGIB/SSBB, and AI/algorithmic reading. Reference lists and forward citations of the included studies and significant reviews were manually searched; related authors were contacted once for any missing management endpoints. 

Data Extraction 

**Figure 1:**
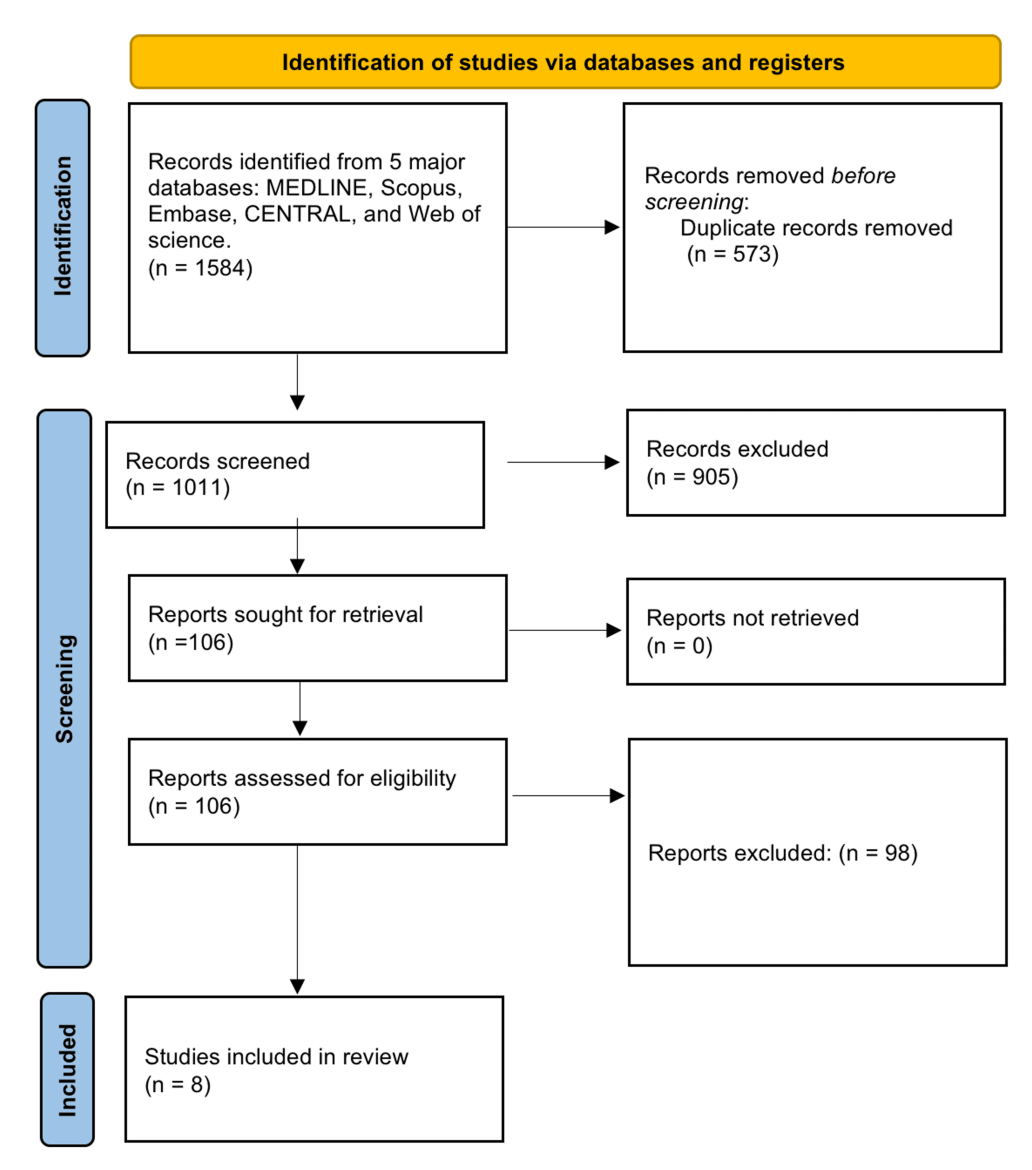
PRISMA flow diagram PRISMA: Preferred Reporting Items for Systematic Reviews and Meta-Analyses

The study selection process was conducted by two researchers (first by title/abstract, followed by full text), with a third reviewer resolving any discrepancies; reasons for exclusion at the full text stage were documented and summarized in the PRISMA flow diagram (Figure 1). For overlapping datasets, the most comprehensive peer-reviewed report was preserved, while supplementary reports were utilized to enhance techniques or outcomes. Two reviewers independently extracted study characteristics (design/tier, setting, platform, AI/algorithm type, reader expertise/blinding, indication mix, definitions of clinically relevant lesions), predetermined outcomes (including time frames), and funding/conflict of interest; discrepancies were resolved through consensus.

Risk of Bias 

The risk of bias was evaluated independently by two reviewers using RoB 2 (Cochrane, London, United Kingdom) for randomized or quasi-experimental trials and Risk Of Bias In Non-randomized Studies - of Interventions (ROBINS‑I) for non-randomized research, both prospective and retrospective. In paired re-reading designs, particular emphasis was placed on order effects (the sequence of reading modes), reader blinding, washout intervals, and selective reporting. Judgments on risk of bias affected the narrative weighing of evidence and imposed sensitivity restrictions, such as the exclusion of studies with substantial or critical risk of bias. 

Due to the heterogeneity in AI systems, platforms, designs (paired versus parallel), and outcome definitions, coupled with inadequate reporting of management endpoints, a meta-analysis was not conducted. Subsequent to SWiM, research was categorized a priori by evidence tier, outcome family (management-direct versus surrogate), AI type (deep-learning CADe versus frame-ranking/rapid modes), and platform (PillCam™ (Medtronic plc, Minneapolis, Minnesota, United Kingdom), MiroCam® (IntroMedic Co., Ltd., Seoul, Republic of Korea), NaviCam® (AnX Robotica Corp, Plano, Texas, United States), and OMOM capsule endoscopy (Jianshan Science and Technology (Group) Co., Ltd, Chongqing, China). 

Certainty of Evidence

The certainty of evidence was assessed using a narrative GRADE (Grading of Recommendations Assessment, Development and Evaluation) methodology for each outcome family, with possible downgrades due to risk of bias, inconsistency (conflicting effect directions), indirectness (heterogeneous cohorts lacking OGIB-specific estimates; dependence on surrogate outcomes), and imprecision (limited sample sizes). Summary-of-findings tables prioritized management outcomes, when available, followed by surrogate measures. All protocol revisions before data extraction, including the implementation of the two-tier architecture and the assignment of surrogate co-primary outcomes, were recorded to ensure transparency.

Results

Study design and attributes that incorporated eight comparative studies across four capsule platforms (PillCam, MiroCam, NaviCam, OMOM) and two levels of evidence (Tier 1 prospective/controlled: four studies [3-6]; Tier 2 paired retrospective: four studies [7-10]). Seven studies provided analyzable sample sizes (n = 679 in total) [3-5,7-10]; Beg et al. did not disclose an explicit sample size (N), only percentages and mean age data [8]. The indications primarily involved obscure or suspected small-bowel bleeding (OGIB/SSBB), with certain groups exclusively exhibiting OGIB or overt bleeding, while others were at least 98% focused on bleeding. Four investigations were performed and compare a person-in-the-loop AI/algorithmic reading mode with traditional human reading. No patient-level management endpoints were measured (Table 1).

**Table 1:** General characteristics of the included studies SBCE: small-bowel capsule endoscopy; OGIB: obscure gastrointestinal bleeding; SSBB: suspected small-bowel bleeding; SR: standard reading; EV: Express View; CADe: computer-aided detection; NR: not reported. *This study did not disclose an explicit sample size (N), only percentages and mean age data

Study (year)	Setting	Design & Evidence Tier	N (analyzed)	Indication(s)	SBCE platform	AI / algorithmic aid	Comparator	Reader blinding / order	Outcomes captured (as per protocol)	Management outcomes reported
Spada et al., 2024 [3]	14 European centers (Italy, Sweden, Spain, Germany, UK, Hungary, France)	Prospective, multicenter, paired second reading; Tier 1	133 (of 137 enrolled)	100% suspected small-bowel bleeding after negative EGD & colonoscopy	NaviCam SB (Ankon/AnX)	ProScan deep-learning CADe (human-in-the-loop, AI-selected frames)	Standard full-video human reading	Blinded second reading; order: Standard → AI-assisted	Per-patient diagnostic yield (P1/P2); reading time	No
Piccirelli et al., 2022 [4]	6 Italian centers	Prospective, multicenter, paired re-read; Tier 1	126	SSBB 98.4% (occult 78.6%; overt 19.8%); suspected neoplasia 1.6%	MiroCam 1200 (IntroMedic)	Express View v3 (ML-based rapid mode)	Standard full-video human reading	Blinded cross-center paired reading; order: SR → EV	Per-patient/per-lesion accuracy; reading time (median 71→13 min)	No
Saurin et al., 2018 [5]	10 French centers	Prospective, multicenter, paired re-read; Tier 1	83	100% OGIB	MiroCam (IntroMedic)	Express View (earlier generation)	Standard reading	Paired reads across centers; consensus reference; order reported	Sensitivity vs consensus; reading time (≈40→20 min)	No
Beg et al., 2020 [6]	9 European centers	Prospective, randomized reading allocation; Tier 1	NR*	71.8% anemia/GI-bleeding among indications	EndoCapsule (Olympus)	Omni Mode (algorithmic frame de-duplication/triage)	Standard reading	Randomized allocation of reading mode to second center	Reading time (mean 42.5 min SR; −24.6 min with Omni); lesion detection (no loss of clinical accuracy)	No
Giordano et al., 2023 [7]	Single center (Hospital Clínic Barcelona)	Retrospective paired re-read; Tier 2	111	100% overt SBB; early CE ≤14 days	PillCam SB3 (Medtronic)	TOP100 (AI-based frame ranking)	Standard reading	Paired blinded readers; SR vs TOP100	Per-patient diagnostic accuracy (P2); reading time (median 23→1.9 min)	No
Arieira et al., 2019 [8]	Single center (Portugal)	Retrospective paired re-read; Tier 2	97	Suspected SBB (mostly occult)	PillCam (Medtronic)	TOP100	Standard reading	Paired protocol	Detection of active bleeding/angiodysplasias; preview/triage utility; (reading time rarely reported)	No
Gomes et al., 2020 [9]	Single center (Portugal)	Retrospective cohort (paired against conventional as reference); Tier 2	89	OGIB with positive CE	MiroCam (IntroMedic)	Express View	Standard reading as reference	Paired comparison to conventional findings	Per-patient sensitivity for clinically significant lesions (83.1%); accuracy 91%	No
O’Hara and McNamara, 2023 [10]	Single center (Ireland)	Retrospective paired re-read; Tier 2	40	50% suspected SBB (rest Crohn’s/polyp surveillance)	OMOM (Jinshan)	OMOM AI model (validated CADe)	Standard reading	Same-video paired analysis	Per-lesion capture; reading time (mean 29.7→2.3 min)	No

Operational Effect (Main Process Result)

In studies that directly assessed interpretation time, AI assistance consistently and significantly reduced reading duration, yielding absolute time savings of approximately 20-58 minutes per study and relative reductions of about 50-92%, resulting in throughput multipliers ranging from approximately 2× to 13× and liberating roughly 33-97 reader-hours per 100 examinations. This tendency persisted across platforms and AI categories, encompassing deep-learning CADe (ProScan, OMOM‑AI) and frame-ranking/rapid modes (TOP100, ExpressView, Omni) (Table 2).

**Table 2:** Operational impact and throughput (reading-time–derived) AI: artificial intelligence (AI-assisted reading); Std: standard reading (conventional human reading); CE: capsule endoscopy; SB: small bowel; SB3: PillCam Small Bowel 3; DL: deep learning; CADe: computer-aided detection; DL-CADe: deep-learning computer-aided detection; EV: ExpressView (MiroCam rapid review mode); TOP100: frame-ranking preview mode (PillCam); Omni: Omni Mode (EndoCapsule); ProScan: Navicam ProScan (deep-learning detection suite); OMOM-AI: OMOM platform AI-assisted reading; min: minutes; h: hours; Δ: absolute difference; ×: fold-change; CE per 8-h day: number of capsule endoscopies a single reader can complete in an eight-hour workday.

Study	Tier	Platform + AI mode	Standard time (min)	AI time (min)	Time saved (min)	% reduction	Reader-hours freed /100 CE	Throughput (Std→AI, ×)	CE per 8-h day (Std → AI)
Spada et al. ,2024 [3]	1	NaviCam SB + ProScan (DL-CADe)	33.7	3.8	29.9	88.7%	49.8 h	8.87×	14.2 → 126.3
Piccirelli et al., 2022 [4]	1	MiroCam 1200 + ExpressView v3	71.0	13.0	58.0	81.7%	96.7 h	5.46×	6.8 → 36.9
Saurin et al., 2018 [5]	1	MiroCam + ExpressView (earlier gen.)	39.7	19.7	20.0	50.4%	33.3 h	2.02×	12.1 → 24.4
Beg et al. 2020., [6]	1	EndoCapsule + Omni Mode	42.5	17.9	24.6	57.9%	41.0 h	2.37×	11.3 → 26.8
Giordano et al., 2023 [7]	2	PillCam SB3 + TOP100	23.0	1.9	21.1	91.7%	35.2 h	12.11×	20.9 → 252.6
O’Hara et al. 2023 [10]	2	OMOM + OMOM-AI	29.7	2.3	27.4	92.3%	45.7 h	12.91×	16.2 → 208.7
Arieira et al., 2019 [8]	2	PillCam + TOP100	23.0	2.0	21.0	91.3%	35.0 h	11.50×	20.9 → 240.0
Gomes et al.,2020 [9]	2	MiroCam + ExpressView	55.4	16.4	39.0	70.5%	65.0 h	3.39×	8.7 → 29.4

Diagnostic Efficacy (Clinically Significant Bleeding-Lesion Yield)

At the individual patient level, AI-assisted interpretation was enhanced with advanced deep-learning systems (e.g., NaviCam + ProScan: +11.3 percentage points for P1/P2 bleeding-relevant lesions), approximately on par with contemporary rapid modes (ExpressView v3), and inferior to a prior generation of rapid modes (early ExpressView: −11.1 pp sensitivity compared to consensus) (Table 3). The overall direction is ambiguous and contingent upon the production of technology, reflecting low-certainty evidence for per-patient bleeding-related yield.

**Table 3:** Decision-impact matrix (diagnostic yield direction + time-saving class → actionable grade) AI: artificial intelligence (AI-assisted reading); Std: standard reading; CE: capsule endoscopy; P1/P2: Saurin classification of bleeding relevance (P2 = highly bleeding-relevant lesion; P1 = possibly bleeding-relevant lesion); Acc.: accuracy; Sens.: sensitivity; DL: deep learning; CADe: computer-aided detection; EV: ExpressView; TOP100: PillCam rapid triage mode; Omni: EndoCapsule Omni Mode; ProScan: Navicam ProScan; OMOM-AI: OMOM artificial intelligence reader; ROB: risk of bias; Tier 1: prospective controlled evidence; Tier 2: retrospective paired evidence; Δ: absolute difference (percentage points); pp: percentage points. Symbols: ▲ AI better; ↔ no clear difference; ▼ AI worse; ? indeterminate.

Study	Tier	Platform + AI mode	Clinically relevant yield vs standard (Δ if stated)	Lesion-class signals (Active bleeding / Angiodysplasia / Ulcer)	Time-saving class	Decision-Impact Grade	ROB
Spada et al.,2024 [3]	1	NaviCam + ProScan	Improved (+11.3 pp per-patient P1/P2)	Not broken down	Very large (≥80%)	A	Moderate
Piccirelli et al., 2022 [4]	1	MiroCam + ExpressView v3	≈ Standard (accuracy vs consensus)	Not broken down	Very large	A	Moderate
Saurin et al., 2018 [5]	1	MiroCam + ExpressView (early)	Lower (−11.1 pp sensitivity vs consensus)	Not broken down	Large (50–<80%)	C	Moderate
Beg et al.,2020 [6]	1	EndoCapsule + Omni	Improved (per-lesion accuracy +5 pts)	Not broken down	Large	B	Some concerns
Giordano et al.,2023 [7]	2	PillCam + TOP100	Improved (triage/capture); high P2 sensitivity	↑ / ↑ / →	Very large	B	Moderate
Arieira et al.,2019 [8]	2	PillCam + TOP100	Mixed (↑ active bleeding, ↑ angiodysplasia, ↓ ulcers)	↑ / ↑ / ↓	Larger	B (mixed)	Moderate
Gomes et al.,2020 [9]	2	MiroCam + ExpressView	Comparable (positive-cases cohort, sensitivity 83.1%)	Not broken down	Large	B	Serious
O’Hara and Mc Namara, 2023 [10]	2	OMOM + OMOM-AI	Improved (per-lesion capture +11.9 pts; diagnosis concordant)	Not broken down	Very large	B	Moderate

Decision-Impact Synthesis (Yield × Duration)

When operational gains were combined with yield direction, two out of eight studies (25%) achieved a stringent Grade A profile (yield ≥ standard and ≥80% time reduction) [3,4], five out of eight (63%) attained Grade B (yield ≥/≈ standard with ≥50% time reduction or mixed lesion-class signals) [6-10], and one out of eight (12%) received Grade C (yield < standard despite time savings) [5]. This matrix demonstrates that AI readers exhibit operational superiority in nearly all studies and are clinically non-inferior or superior in the majority, with the sole Grade C outlier corresponding to an earlier generation of algorithms (Table 3).

Risk of Bias

According to RoB-2 (Table 4), the sole randomized reading-allocation study exhibited several problems with randomization, measurement, and reporting [6]. Among non-randomized research (Table 5), the majority presented a moderate risk with anticipated paired-design complications (fixed order SR→AI, mode-aware reading), while one study exhibited a substantial risk due to positive-only selection and the use of standard reading as a reference [9].

**Table 4:** RoB 2 (Tier 1 randomized study)

Study (year)	Tool	Overall	Randomization process	Deviations from intended interventions	Missing outcome data	Measurement of the outcome	Selection of the reported result
Beg et al., 2020 [6]	RoB 2	Some concerns	Some concerns	Low	Low	Some concerns	Some concerns

**Table 5:** ROBINS-I (Tier 1 non-randomized and Tier 2 retrospective paired studies)

Study (year)	Tool	Overall	Confounding	Selection of participants	Classification of interventions	Deviations from intended interventions	Missing data	Measurement of outcomes	Selection of the reported result	Order effect	Reader blinding	Washout
Spada et al., 2024 [3]	ROBINS-I	Moderate	Moderate	Low	Low	Low	Low	Moderate	Low	Fixed	Yes	N/A
Piccirelli et al., 2022 [4]	ROBINS-I	Moderate	Moderate	Low	Low	Low	Low	Low	Moderate	Fixed	Yes	N/A
Saurin et al., 2018 [5]	ROBINS-I	Moderate	Moderate	Low	Low	Low	Low	Low	Moderate	Fixed	Yes	N/A
Giordano et al., 2023 [7]	ROBINS-I	Moderate	Moderate	Low	Low	Low	Low	Low	Moderate	Fixed	Yes	N/A
Arieira et al., 2019 [8]	ROBINS-I	Moderate	Moderate	Low	Low	Low	Low	Low	Moderate	Fixed	Yes	Unclear
Gomes et al., 2020 9]	ROBINS-I	Serious	Serious	Serious	Low	Low	No information	Serious	Moderate	N/A	Unclear	N/A
O’Hara and McNamara, 2023 [10]	ROBINS-I	Moderate	Moderate	Low	Low	Low	Low	Moderate	Moderate	Fixed	No	Yes

Certainty of Evidence

The summary of findings affirms: (i) the absence of comparative evidence for management-directed outcomes (time-to-diagnosis/intervention, transfusions, therapeutic yield of BAE/IR, rebleeding/readmission, costs beyond reading), (ii) moderate-certainty evidence indicating substantial and consistent reductions in reading time, and (iii) low-certainty evidence regarding clinically significant yield per patient, with a generational effect favoring newer deep-learning AI (Table 6).

**Table 6:** Certainty of Evidence (GRADE)

Outcome (prioritized)	Comparative studies (n)	Participants (approx.)	Effect (study-level summary)	Certainty (GRADE)	Main reasons for rating
Management-direct outcomes (time-to-diagnosis/intervention; transfusion; therapeutic yield of BAE/IR; rebleeding/readmission; costs beyond reading)	0	—	No comparative evidence identified in the included studies.	— (no data)	None of the included studies measured these endpoints.
Reading time per study (min)	6	≥493	Consistent, large reduction with AI across platforms; typical absolute saving 20–58 min; median relative reduction ≈85%; throughput increase ~2×–13×.	Moderate	Risk of bias (paired/unblinded designs) −1; consistent, very large effects across settings (no downgrade for inconsistency); indirectness not serious; imprecision not serious; publication bias unclear.
Per-patient yield of clinically relevant bleeding lesions (e.g., Saurin P2/bleeding-relevant)	3	342	Mixed: improved with newer DL-CADe (+11.3 pp), ≈standard with modern rapid mode, lower with early rapid mode (−11.1 pp).	Low	Some risk of bias; inconsistency across AI generations; modest sample sizes/heterogeneous metrics.
Inter-reader reliability (κ), recalled frames, workload/usability	Few, descriptive	—	Recalled/flagged frame reduction substantial in rapid modes (~95% reduction in images reviewed); κ and workload rarely reported comparatively.	Very low	Sparse/heterogeneous reporting; indirectness and imprecision; potential reporting bias.

The Implications for Current Practice

In services assessing adoption, operational excellence was significant; AI help can enhance throughput by around 2-13 times while preserving at least equivalent detection of bleeding-related issues in most contexts, particularly with modern deep-learning technologies (Tables 2, 3). Nonetheless, as no study provided evidence of enhancement in patient-level outcomes, these procedural benefits should be executed with prospective monitoring of subsequent decisions and results to bridge the evidence gap (Table 6).

Relevance of the Context

The results are applicable to OGIB/SSBB populations primarily following negative bidirectional endoscopy, as well as to both multicentre and single-centre procedures, and are consistent across common capsules and software (PillCam, MiroCam, NaviCam, OMOM; AI modes including ProScan, TOP100, ExpressView v3, OMOM‑AI, Omni) (Table 1).

Discussion 

SBCE is the primary diagnostic test for adults with unclear or suspected small-bowel bleeding after negative bidirectional endoscopy. Professional societies recommend SBCE first, followed by device-assisted enteroscopy for targeted therapy as needed [1,2,11-13]. Our analysis shows that AI-assisted, human-in-the-loop reading improves efficiency while maintaining or even enhancing clinically relevant bleeding-lesion detection compared to traditional reading methods. This trend is consistent with guideline targets (quick clarification of bleeding cause and streamlined triage), but it also reveals a continuing evidence gap: no comparable study to date has assessed patient-level outcomes such as time-to-therapy, transfusion needs, rebleeding, or expenditures.

The Significance of Speed in OGIB

The timing influences the observations of SBCE and the speed at which care can transition to therapy. Timely capsule deployment, particularly in cases of overt bleeding, enhances lesion identification and enables more effective subsequent care; delays increase the danger of regression of stigmata or the oversight of intermittent hemorrhage [14-16]. By reducing reading time without compromising diagnostic accuracy, AI-assisted workflows are set to decrease the duration from data collection to treatment decisions, an area where OGIB pathways are most vulnerable to delays [14-16].

Contributions from the AI Capsule Endoscopy Literature

Over the past decade, deep-learning systems have achieved gastroenterologist-level performance in frame-level categorization for SBCE and can reliably prioritize clinically significant images [17]. Convolutional neural networks (CNNs) effectively identify core bleeding-relevant targets-ulcers/erosions and angioectasias, on actual capsule images, providing a viable foundation for first triage [18-20]. Supplementary bleeding indicators, such as blood/haematin, are assimilated efficiently, facilitating end-to-end workflows that mitigate human tiredness and overlooked discoveries [21].

In addition to single-lesion tasks, multiclass systems may analyze complete movies for various anomalies and have advanced to practical, prospective validations, hence enhancing the translational readiness of AI-assisted review [22,23]. Recent studies categorize protruding lesions and assess their hemorrhagic potential-attributes that may connect identification to actionable outcomes when integrated into therapeutic protocols [24,25]. Concurrent progress in inflammatory conditions (e.g., Crohn’s ulcers and strictures) demonstrates a transferable technical proficiency across small-bowel phenotypes, enhancing assurance that bleeding-related procedures are not anomalies but integral to a wider performance spectrum [26,27].

Evaluations and Anticipated Operational Advantages

Methodological overviews and meta-analyses of AI in SBCE consistently convey two key points: AI diminishes the number of frames necessitating human evaluation and reduces interpretation duration, all while preserving (or enhancing) lesion identification in real-world datasets [28,29]. In the context of conventional SBCE workloads, typically marked by lengthy videos, inconsistent preparation, and significant incomplete/retention rates, the influence of reliable triage on capacity is clearly apparent for services aiming to diminish backlogs and expedite multidisciplinary decisions [30].

Insights From AI-Related Endoscopy

Randomized trials of CADe in colonoscopy consistently enhance adenoma detection rates without compromising workflow, indicating that prospectively integrated AI can advance quality metrics in routine practice [31-33]. Although OGIB outcomes vary from adenoma measurements, these trials exemplify the comprehensive, patient-centered assessment that the SBCE field should replicate to translate time savings into tangible clinical advantages.

Technological Trajectory and Platform Considerations

SBCE is advancing with advancements in capsule optics, localization, and active locomotion, all of which will engage with AI readers in the forthcoming years [34]. Strategies for image augmentation are being investigated for capsule endoscopy, potentially improving AI-assisted detection in conditions of deficient visibility, indicating synergistic results when integrated with contemporary networks [35]. These paths render it increasingly probable that AI-assisted reading will emerge as the standard interface of SBCE rather than a supplementary feature.

Methodological Rigor and Documentation

This review was organized and presented in accordance with PRISMA 2020 and SWiM guidelines, assessed certainty using GRADE, and evaluated bias through RoB 2 and ROBINS-I, decisions that are consistent with current standards for transparent evidence synthesis in diverse, non-pooled literatures [36-40]. In the future, clinical AI trials in SBCE should prospectively register protocols and adhere to CONSORT‑AI (Consolidated Standards of Reporting Trials-Artificial Intelligence) and SPIRIT‑AI (Standard Protocol Items: Recommendations for Interventional Trials-Artificial Intelligence), utilizing DECIDE‑AI (Developmental and Exploratory Clinical Investigations of DEcision-support systems driven by Artificial Intelligence) for preliminary evaluations and TRIPOD‑AI (Transparent Reporting of a multivariable prediction model for Individual Prognosis Or Diagnosis-Artificial Intelligence)/PROBAST (Prediction model Risk Of Bias ASsessment Tool) for the reporting and assessment of prediction models; these frameworks collectively mitigate unnecessary biases and elucidate the integration of AI into human workflows [41-45]. Diagnostic accuracy studies must comply with Standards for Reporting of Diagnostic Accuracy (STARD) to guarantee that sensitivity and specificity assertions result in dependable, clinical-grade outcomes [46].

Healthcare systems are transitioning to "high-performance medicine," wherein human knowledge is enhanced by validated digital tools to provide expedited, safer, and more egalitarian care [47]. In OGIB, the future depends on matching SBCE outcomes with the values of doctors and patients: earlier targeted therapy, reduced transfusions, fewer rebleeds, and decreased resource utilization. Standardizing clinically pertinent lesion definitions, such as the P0/P1/P2 classification, remains crucial to ensure that advancements in detection correlate with decisions and, eventually, with significant results [48].

## Conclusions

AI-assisted capsule endoscopy demonstrates operational superiority over traditional reading methods in cases of obscure or suspected small-bowel bleeding, decreasing interpretation time, and increasing throughput by up to 13 times, while preserving, and in some instances enhancing, diagnostic yield with contemporary deep-learning systems. However, no research has evaluated patient-specific outcomes, including time-to-therapy, transfusions, or rebleeding. The present certainty is moderate about efficiency, low concerning yield, and nonexistent for therapeutic results. AI can currently be implemented as a triage-first, verify-second workflow to alleviate reporting backlogs; however, future trials must assess patient-centered and cost results to see if these operational improvements result in significant therapeutic benefits.
